# Malleability of spatial skills: bridging developmental psychology and toy design for joyful STEAM development

**DOI:** 10.3389/fpsyg.2023.1137003

**Published:** 2023-09-12

**Authors:** Çiğdem İrem İleri, Melisa Erşan, Duru Kalaça, Aykut Coşkun, Tilbe Göksun, Aylin C. Küntay

**Affiliations:** ^1^Department of Psychology, Koç University, Istanbul, Türkiye; ^2^Department of Design and Technology, Parsons School of Design, The New School New York, NY, United States; ^3^Department of Media and Visual Arts, Koç University, Istanbul, Türkiye; ^4^Koç University-Arçelik Research Center for Creative Industries, Koç University, Istanbul, Türkiye

**Keywords:** informal STEAM development, toy design, construction toys, mental rotation, mental folding, perspective taking, spatial cognition

## Abstract

Previous research has established that advances in spatial cognition predict STEAM success, and construction toys provide ample opportunities to foster spatial cognition. Despite various construction toy designs in the market, mostly brick-shaped building blocks are used in spatial cognition research. This group of toys is known to enhance mental rotation; however, mental rotation is not the only way to comprehend the environment three-dimensionally. More specifically, mental folding and perspective taking training have not received enough attention as they can also be enhanced with the construction toys, which are framed based on the 2×2 classification of spatial skills (intrinsic-static, intrinsic-dynamic, extrinsic-static, extrinsic-dynamic). To address these gaps, we compile evidence from *both* developmental psychology *and* toy design fields to show the central role played by mental folding and perspective taking skills as well as the importance of the variety in toy designs. The review was conducted systematically by searching peer reviewed design and psychology journals and conference proceedings. We suggest that, over and above their physical properties, construction toys offer affordances to elicit spatial language, gesture, and narrative among child-caregiver dyads. These interactions are essential for the development of spatial skills in both children and their caregivers. As developmental psychology and toy design fields are two domains that can contribute to the purpose of developing construction toys to boost spatial skills, we put forward six recommendations to bridge the current gaps between these fields. Consequently, new toy designs and empirical evidence regarding malleability of different spatial skills can contribute to the informal STEAM development.

## 1. Introduction

The acronym STEAM (Science, Technology, Engineering, Arts, and Mathematics) is usually collocated with the terms “learning” or “education.” However, these terms often have formal and technical connotations. On the other hand, informal toy play sessions provide an alternative context to incorporate STEAM improvement into the daily routine (Bergen, 2009; Toub et al., 2019; Martin and Murphy, 2022) since out-of school activities are just as important as in-school activities for STEAM development (Gözüm et al., 2022), and children spend a considerable amount of time playing with toys outside of school (Giddings and Halverson, 1981; Halpern et al., 2007; Bekker et al., 2009). Research demonstrates that playing with construction toys, which consist of units assembled in multiple configurations (Brosnan, 1998; Stannard et al., 2001; Weller et al., 2008), predicts achievement in STEAM-related disciplines (Wai et al., 2009; Trawick-Smith et al., 2016; Borriello and Liben, 2017). The primary mediatory factor in this relationship is spatial cognition, which refers to the ability to interact with the vicinity in a three-dimensional way, physically or mentally (Vasilyeva and Lourenco, 2012; Newcombe et al., 2013; Bower et al., 2020a). Children employ spatial cognition in many daily activities, such as tool use, games, route finding, and school-related tasks.

Developmental psychology and toy design are the two fields relevant to using toys for improving spatial cognition and STEAM success. However, these fields are currently not well connected. On the one hand, developmental psychology studies are conducted mainly with a limited variety of toys (e.g., building blocks); alternative toy designs are not considered for enhancing spatial development, yet their affordances may contribute to different aspects of spatial cognition. Although there were some attempts to conduct research on the different construction toys, they were shadowed with methodological concerns. For instance, Vander Heyden et al. (2017) assessed the improvement in the various spatial skills with a pre- and post-test intervention that involved various toys; however, the toys were not distributed systematically in the training procedure. Participants played with several toys in each training session and their cumulative effect was tested at the post-test phase. Hence, the separate effect of each training session and each toy design on the post-test results was obscured. In another study, Ralph et al. (2020), magna tiles toy was used rather than typical building blocks. Yet, there was no emphasis on this toy’s affordances (i.e., how its physical attributes support spatial cognition). It was chosen solely as a context to elicit spatial play by mother and child dyads.

On the other hand, toy design research rarely considers evidence from developmental psychology regarding toy development and child interaction. The ones that involve psychology theories sometimes lack depth in their conceptual definitions. For instance, Geurts et al. (2014) developed digital cubes to improve perspective taking skill however the term was defined in a limited way as detecting right and left of another agent. In another study, Rigo et al. (2016) criticized the repetitive cubicle design of building blocks in the market, and proposed an alternative design that consists of various polyhedrons. However, they did not provide a rationale to their new design in relation to development of spatial skills.

Instances above demonstrate the lack of a dialog between developmental psychology and toy design literatures. The current review aims to highlight and strengthen this connection, which is a first attempt for both lines of literature. While illustrating the gaps, first the existing evidence on the malleability of three different spatial skills (i.e., mental rotation, mental folding, and perspective taking) will be presented. Then some toy design examples will be shared, in relation to their potential to foster these three spatial skills. Last, six recommendations will be provided for future research to highlight what can be done further regarding toy design. Also a benchmark of construction toys will be presented based on their contribution to spatial skill development. The upshot of this review is to combine the perspectives of developmental psychology and design research to address how to diversify the spatial affordance of toys for child-caregiver dyads. Although some existing studies attempt to bridge the psychology and the design literatures (Hekler et al., 2013; Beşevli et al., 2022), no study so far has focused on the spatial cognition domain in relation to toy design.

## 2. State of the art

Playing with spatial toys such as blocks and puzzles offers opportunities for exploring different object orientations and viewer perspectives (Casey et al., 2008; Pirrone et al., 2015; Vander Heyden et al., 2017). Similar mental exercises are required for STEAM fields (Hinze et al., 2013; Uttal et al., 2013b; Polinsky et al., 2022); consequently, playing with those toys facilitates STEAM development (Wolfgang et al., 2003; Hanline et al., 2010; Taylor and Hutton, 2013).

Three spatial skills are potentially related to STEAM success. First, *mental rotation,* which refers to changing the orientation of an object’s mental representation at a certain angle (Shepard and Metzler, 1971; Hawes et al., 2015; Lauer et al., 2015). Second, *mental folding,* which stands for changing the physical properties of an object while moving it in a given space, for example, transforming a two-dimensional paper into a three-dimensional structure (Atit et al., 2013; Harris et al., 2013b; Burte et al., 2017). Third, *perspective taking,* which refers to the ability to see a scene from another point of view (Kessler and Rutherford, 2010; Erle and Topolinski, 2015; Gunia et al., 2021). Previous studies have mainly investigated mental rotation training, while mental folding and perspective taking skills have been largely overlooked (Cherney et al., 2014; Newcombe and Shipley, 2014; Vander Heyden et al., 2017). Thus, training in those two spatial skills needs attention considering their contribution to STEAM development (Mix and Cheng, 2012; Newcombe, 2017; Hodgkiss et al., 2018).

While it is expected that developmental psychology literature should use and compare various toy designs for their impact on different spatial skills, design studies should also incorporate theoretical frameworks of developmental psychology into the toy design process. However, these frameworks may not be readily available for toy designers to access and interpret (Hall et al., 2022). Furthermore, the reasons why toy producers add certain features to their designs and what particular points they consider while designing construction toys are not immediately transparent, even though there may in fact be several theoretical foundations employed in the current toy designs (DeCortin, 2015). Therefore, enhancing the spatial characteristics of the construction toys is an important step in bridging the gaps observed between theory and practice (Hekler et al., 2013; Borriello and Liben, 2017; Yang et al., 2020). Through this review paper, we aim to provide the following recommendations to the design field by revisiting both strands of literature:

1. Include mental folding components.
2. Design large-scale toys to facilitate perspective taking skill.
3. Consider the entire user experience, in addition to the physical properties of the toys.
4. Embrace multiple personas (i.e., adults and children).
5. Add features to elicit spatial language, narrative, and gesture use.
6. Avoid using extremely themed products.

## 3. Method

A literature review was conducted systematically to assemble various research studies written about malleability of spatial skills, STEAM education, and toy design between the years 1969 and 2022. In this review, we used both expansive databases like Google Scholar, Elsevier, ProQuest, PubMed and EBSCO, and domain specific databases like PsycInfo and ACM digital library. The keywords used to explore relevant articles were “malleability,” “spatial cognition,” “perspective taking,” “mental rotation,” “mental folding,” “construction toys,” “building blocks,” “spatial training,” “spatial intervention,” “spatial input,” and “informal STEAM education.” We used the handles “AND,” to affiliate search terms between each category, and “OR,” to connect search terms within each category.

All articles examined in the process of the literature review were analyzed based on their abstracts by the first three authors. Then based on the relevance of each article to the current concept of this paper, articles were either removed from the reference list or kept to be further analyzed to support the research of this paper. While the articles were being reviewed, their reference lists were also examined to expand the scope of the literature review through snowballing.

The publications included in the literature search would be considered suitable if they met the following criterion: (a) articles focusing exclusively on malleability of spatial skills or construction toy design, (b) papers only published in English, and (c) papers that are published in peer-reviewed journals. Papers were excluded based on the following criteria: (a) works that are not accessible through the databases, (b) works that did not address the relationship demonstrated in the objectives of this paper, which are construction toys and spatial skill development, (c) any unpublished data, and (d) and short communications, and editorials.

## 4. Spatial cognition

Spatial cognition is an essential ability for many species as it enables individuals to understand the three-dimensional world better. Skills that make up spatial cognition reveal themselves in two ways: Tool making and navigating in the environment (Ehrlich et al., 2006; Morganti et al., 2009; Newcombe et al., 2013). Thus, spatial cognition directly connects to daily tasks like understanding a map or organizing a wardrobe (Vasilyeva and Lourenco, 2012; Meneghetti et al., 2015; Bower et al., 2020a). Aside from these uses, spatial skills are related to school readiness (Verdine et al., 2014) and numerical cognition (Newcombe et al., 2015), consequently predicting achievement in STEAM disciplines (Wai et al., 2009; Taylor and Hutton, 2013; Uttal et al., 2013b).

The conceptual scope of spatial cognition must be clarified since various definitions exist in the literature and a common framework is yet to be established (Resnick and Shipley, 2013; Newcombe and Shipley, 2014; Mix et al., 2018). A comprehensive and empirically tested theory, which is supported by neural (Creem et al., 2001; Wraga et al., 2005; Lambrey et al., 2011) and behavioral findings from different age groups (Taylor and Hutton, 2013; Vander Heyden et al., 2017; Hodgkiss et al., 2018), classifies spatial skills through a 2×2 matrix. This matrix is based on the mental representations’ static/dynamic and intrinsic/extrinsic properties (Uttal et al., 2013a; Yang et al., 2020; Bower et al., 2020b) (see Table 1).

**Table 1:** 2×2 classification of spatial skills and examples [adapted from Uttal et al. (2013a)].

Spatial skill	Definition	Example
Intrinsic-static	Apprehending objects, paths, or spatial placements over distracting background information	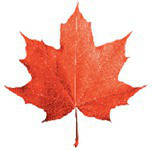
Intrinsic-dynamic	Bringing objects into more complex placements, mentally rotating objects or transforming from 2D to 3D	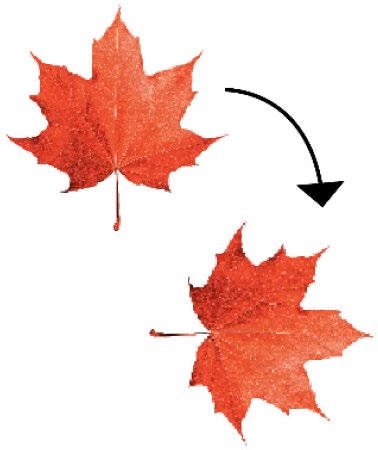
Extrinsic-static	Recognizing and apprehending spatial principles relatively to other objects such as horizontality and verticality	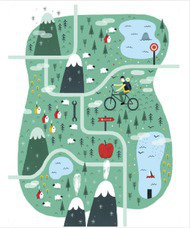
Extrinsic-dynamic	Mentally representing an environment in its full shape from various perspectives	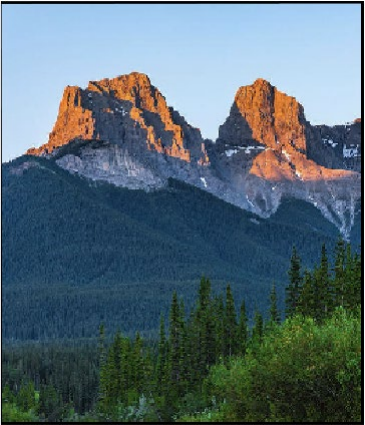

Static spatial cognition allows individuals to interpret non-moving objects, while dynamic spatial cognition enables them to follow changing stimuli. Research shows that static and dynamic spatial cognition have different underlying mechanisms (Kozhevnikov et al., 2002, 2005). The other axis of the 2×2 matrix includes intrinsic and extrinsic spatial representations. Intrinsic spatial cognition refers to understanding within object relationships, and it is a key skill for tool use (Harris et al., 2013a; Newcombe and Shipley, 2014; Frick, 2018). Extrinsic spatial cognition refers to understanding the relationship between objects, which is necessary for navigation (Kinach, 2012; Atit et al., 2013; Newcombe et al., 2013). Research indicates that extrinsic and intrinsic spatial skills follow different neural pathways (Chatterjee, 2008; Li et al., 2019; Gunia et al., 2021), and have separate mechanisms (Huttenlocher and Presson, 1973; Hegarty and Waller, 2004; Newcombe et al., 2013; Hodgkiss et al., 2018). Vander Heyden et al. (2017) explain this mechanism via different strategies used by the participants for mental transformation and perspective taking tasks: object transformation and viewer transformation, respectively. Through the object transformation strategy, participants tend to mentally change the target object’s orientation without changing their position. On the other hand, during the viewer transformation strategy, participants mentally rotate themselves in a given space and change their frame of reference to perceive an object from a different point of view (Hegarty and Waller, 2005; Harris et al., 2013a; Vander Heyden et al., 2017).

In the current review, we will focus on the informal training tools (i.e., toys) for the two intrinsic dynamic spatial skills: mental rotation and mental folding, and an extrinsic dynamic skill: perspective taking. The literature is rich in examining intrinsic dynamic spatial relations (Frick et al., 2013a; Uttal et al., 2013a; Newcombe and Shipley, 2014). However, existing studies primarily focus on mental rotation; overlooking the mental folding skill (Atit et al., 2013; Harris et al., 2013a; Hilton et al., 2022). Furthermore, there is a lack of training for improving extrinsic dynamic spatial skills (i.e., perspective taking) (Mori and Cigala, 2015; Vander Heyden et al., 2017; Tian et al., 2021). Perspective taking skill is crucial as it is both a spatial and social ability (Shelton et al., 2012; Clements-Stephens et al., 2013; Tarampi et al., 2016) supported by neural findings (Lambrey et al., 2011; Gunia et al., 2021). Socio-communicational tasks such as referential communication (Keysar et al., 2000; Nilsen and Fecica, 2011; Yadollahi et al., 2022), empathy (Erle and Topolinski, 2015), and Theory of Mind demand the utilization of perspective taking (Barnes-Holmes et al., 2004; Tian et al., 2021; Strikwerda-Brown et al., 2022). Studies with developmentally atypical individuals are in line with the social component of perspective taking since children with autism spectrum disorder experience difficulty in visuospatial perspective taking tasks, while their mental rotation skill is intact (Hamilton et al., 2009; Pearson et al., 2013; Cardillo et al., 2020). There may indeed be a connection between perspective taking capacity and STEAM achievement, even though the link is not studied much (Mix and Cheng, 2012; Newcombe, 2017).

### 4.1. Mental rotation

Intrinsic-dynamic skills include rotating, folding, slicing, bending, or any other manipulation of the mental representation of an object (Shepard and Cooper, 1982; Resnick and Shipley, 2013; Baykal et al., 2018). Among these various transformations, *mental rotation* is the prototypical spatial representation (Mix and Cheng, 2012; Frick et al., 2013a; Bruce and Hawes, 2014). This skill is often measured by presenting participants with shapes that have been oriented and rotated at different angles and asking them to identify the target shapes (Shepard and Metzler, 1971; Neuburger et al., 2012; Frick et al., 2013b). This type of mental transformation has been called a “rigid transformation” because no matter how an object is rotated, it will maintain its initial properties, such as the distance between any of its two corners (Atit et al., 2013; Resnick and Shipley, 2013; Harris et al., 2013a).

The capacity to process abstract stimuli is the common mechanism between the well-developed mental rotation skill and higher achievement in the STEAM fields. For instance, students or professionals who engage in chemistry need to comprehend the three-dimensional structures of the molecules from various angles, which is a pretty similar task to mental rotation (Hinze et al., 2013; Resnick and Shipley, 2013; Uttal et al., 2013b). Additionally, tasks in mathematics require similar representations with mental rotation, such as moving or manipulating operants (Cheng and Mix, 2013; Tosto et al., 2014; Pirrone et al., 2015), and in geometry, students or professionals need to be able to reason about form and angle of the shapes, just like in the mental rotation tasks (Kinach, 2012; Mix and Cheng, 2012; Bruce and Hawes, 2014). Hence, there is a strong link between performance in STEAM fields and mental rotation tasks (Wai et al., 2009; Uttal et al., 2013a; Hawes et al., 2019).

Moreover, studies suggest that mental rotation skill can be improved (Sorby, 2009; Cheng and Mix, 2013; Kornkasem and Black, 2015). The value of mental rotation concerning STEAM success, combined with the proposal that it is malleable, signifies a need to research its training methods (Caldera et al., 1999; Toub et al., 2019). A meta-analysis by Uttal et al. (2013a) reveals various methods for improving spatial cognition. One of those methods is to reproduce a vast amount of test items from a traditional mental rotation task and to give some items as a training stimulus while giving the rest as testing items (Wright et al., 2008; Meneghetti et al., 2015; Contreras et al., 2018). This method has the theoretical power to demonstrate that spatial cognition is malleable; however, it receives several criticisms. First, it is not an ecologically valid training because individuals do not face similar spatial problems in daily life (Morganti et al., 2009). Second, it is not a proper way to apply in a practical setting such as school when the goal is actually to improve those skills since the task is exhausting and time-consuming, especially for children (Geurts et al., 2014; Newcombe, 2017).

Playing with construction toys, however, is an exceptional method of enhancing spatial skills by engaging in daily routines. More time spent playing with construction toys such as LEGO®'s, Mega Bloks, etc., positively correlates with higher scores in spatial tasks, even when controlling for general cognitive abilities (Jirout and Newcombe, 2015). Assembling construction toys stimulates the exploration of different object positionings in space; consequently, they provide an opportunity to practice mental rotation (Casey et al., 2008; Pirrone et al., 2015; Polinsky et al., 2022). Furthermore, those toys are made from units, and as children build various compositions with them, they create complete mental representations of the units. In the next section, a couple of toy examples that foster mental rotation will be introduced.

### 4.2. Toy examples for enhancing mental rotation

#### 4.2.1. Traditional toys

LEGO-type building blocks are vastly known for contributing to development of mental rotation skills. Their key affordance is the modularity of the construction units to reassemble multiple times (Brosnan, 1998). In this way, they aid in exploring various configurations in a defined space. Indeed, several toy designs may facilitate mental rotation skills apart from LEGO^®^. Each of these toys has similar modular systems that signify how and in which direction the construction play should be structured, yet affordances vary based on their elements’ shape, scope, and scale (see Appendix A). Despite their differences, with all the toys, the play interaction requires the key action of assembling within two planes, in the x-axis or y-axis, which creates a rigid transformation during the play experience (Atit et al., 2013; Resnick and Shipley, 2013; Harris et al., 2013a).

**Figure 1:**
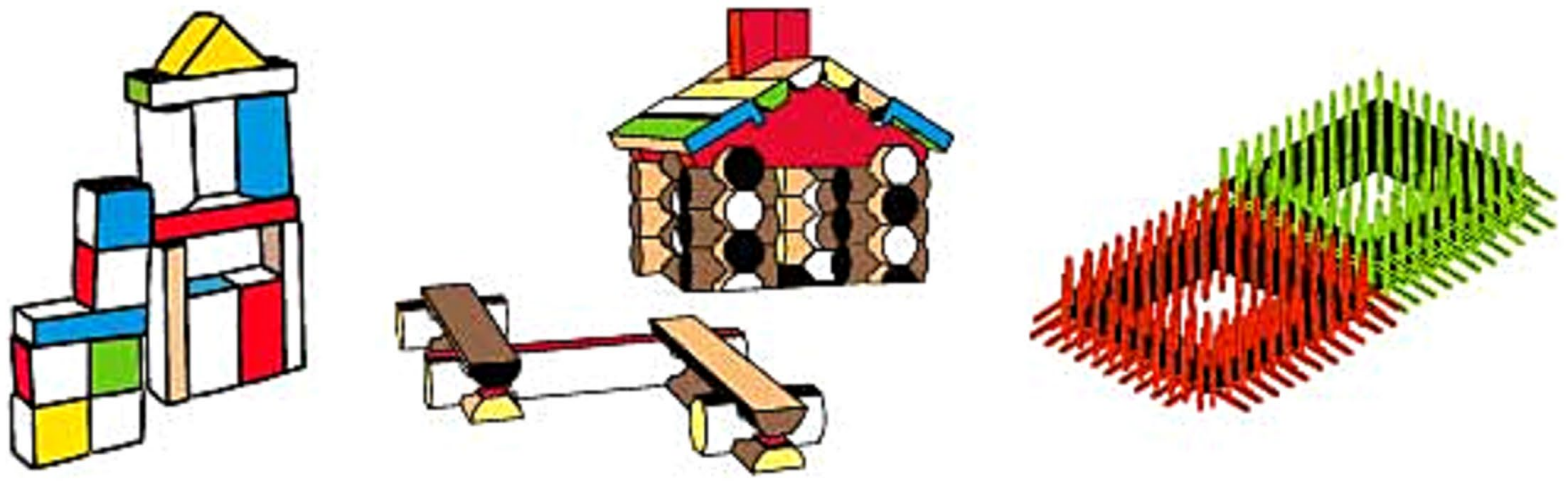
Toy examples to foster mental rotation (left to right; Unit Blocks, Lincoln Logs, Bristle Block).

**Figure 2:**
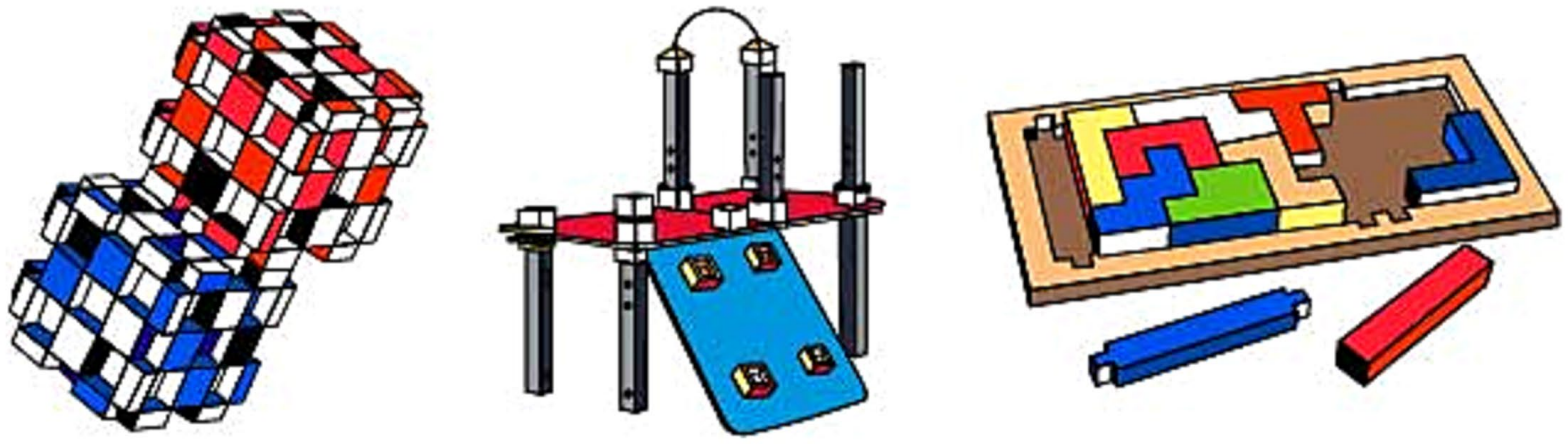
Toy examples to foster mental rotation cont (left to right; KüpTak, Learning Resources City Engineering, Katamino).

Toys like Unit Blocks, Montessori Wooden Blocks, Lincoln Logs, Bristle Blocks, KÜP-TAK, Jeujura Wooden Construction Toy, Learning Resources City Engineering, Tangram, and Katamino are a few of the many to highlight within this category (see Figures 1, 2). Those toys are chosen based on market research within established online shopping websites (e.g., Amazon, eBay, etc.). The keywords “construction toy, building block, manipulatives” were used during the market search phase. All these toys can be played by hand and carried around, allowing users to transform their configurations easily. Even though each toy varies regarding the narrative or the form it embodies, they can be grouped together based on their similar affordances (see Appendix A). Unit Blocks, Montessori Wooden Blocks, and Lincoln Logs are composed of primitive-shaped units, and the flat surfaces on each side indicate that units can be stacked on top of each other. Compared to previously introduced toys, one distinct feature of these toys is that they do not have a joint system to assemble pieces together. As a result, the play activity is impacted since, without a joint mechanism, the durability of the structure’s core will be limited, and pursuing taller structures is not feasible. Therefore, each piece can only be rotated or stacked while building. Subsequently, toys like Learning Resources City Engineering and Jeujura Wooden Construction Toy deliver real-life narratives like building a chalet and a construction site. Another key aspect of Learning Resources City Engineering and Jeujura Wooden Construction Toy is that they possess a joint mechanism on the edges of their modular pieces, providing more balanced and durable structures to be built. Bristle Blocks and KÜP-TAK also embody a joint mechanism, while their grips and holes coat each surface of their modular shapes, allowing an increased variability of shape formations. Last, traditional toys like Tangram and Katamino only enable users to play with configurations of objects on a designated two-dimensional surface. Because of this limitation, while the user can alter the placement of each module by rotating, they cannot build additional levels, which limits the expansion of the play experience.

#### 4.2.2. Digital tools

**Figure 3:**
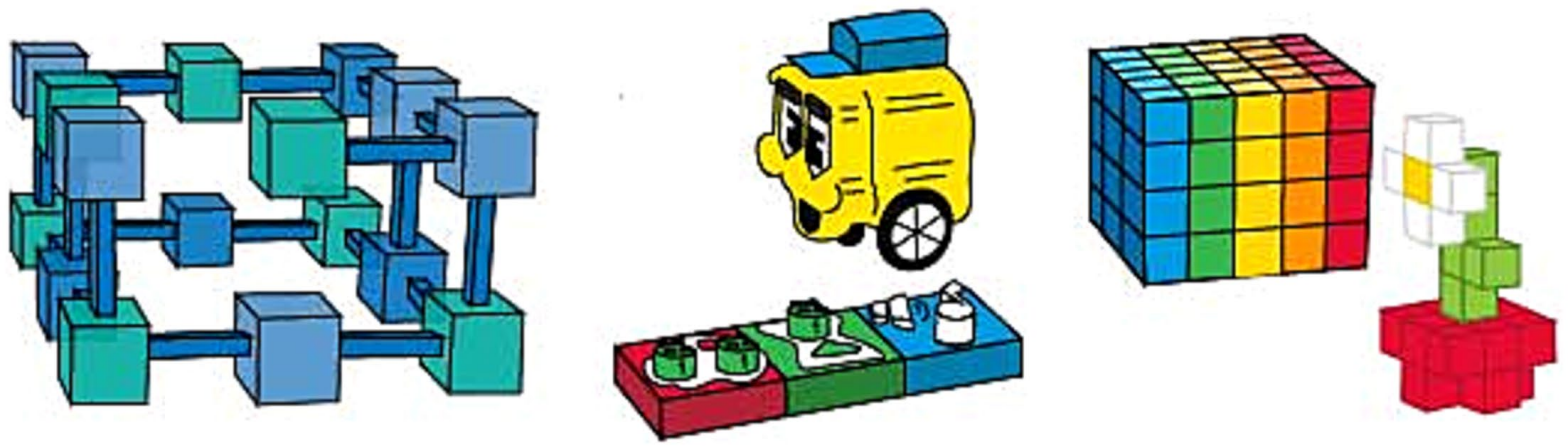
Tangible user interface examples to foster mental rotation (left to right; Boda Blocks, AlgoBrix, Pixio).

Moreover, with the rapid increase in technological toys (Ho et al., 2017; Gözüm and Kandır, 2021; Hall et al., 2022), electronic alternatives for spatial play find a considerable market. For example, Tangible User Interfaces (TUIs), which refer to technologically augmented physical entities (Pires et al., 2019), are studied in design literature due to their potential to support the enhancement of spatial cognition (Baykal et al., 2018), alongside traditional (non-electronic) toys (Zosh et al., 2015; Healey and Mendelsohn, 2018; Hassinger-Das et al., 2021). The most salient benefit of digital tools in spatial skill development is to provide affordances for exploring and formulating spatial representations beyond the direct experience. They enable users to expand their spatial thinking to the digital medium (Pires et al., 2019). Boda Blocks, Algobrix, and Pixio are some examples to review in addition to traditional toys (see Figure 3). Boda Blocks is an experimental TUI created by Buechley and Eisenberg (2007), made up of 16 cubes that light up to be green or blue and that can be arranged in different configurations. Some connectors can be attached to any of the six sides of a cube and can be used to tie the cubes to each other. Only one connector can be attached to each surface. The software accompanying the blocks program displays various dynamic three-dimensional light and color patterns, enabling users to experience spatial features multimodally (Buechley and Eisenberg, 2007). Algobrix is compatible with LEGO^®^ pieces thanks to their similarity in size and affordances. Additionally, the toy enables users to turn their constructions into robots by coding to perform various actions. Last, Pixio comprises 8x8x8 mm magnetic cubes that can be attached on all sides, allowing the creation of abstract shapes, animals, buildings, etc. Its small size makes the units easy to manipulate by hand. Pixio’s unique feature is its expansion to the digital medium through a mobile application scanning the constructions. In this way, the toy provides opportunities for viewing, manipulating, and moving in virtual space, altering numerous physical and digital structures.

### 4.3. Mental folding

Neurological evidence supports that mental rotation and mental folding are distinct (Milivojevic et al., 2003; Harris et al., 2013a), but related skills (Hilton et al., 2022) under the intrinsic dynamic category of spatial cognition. When the mental representation of a shape is folded, unlike in mental rotation, properties of the shape change (Resnick and Shipley, 2013; Hodgkiss et al., 2018; Toub et al., 2019), and an infinite number of new shapes and objects can be created depending on where or how many times the original shape is folded (Atit et al., 2013; Megahed, 2017). These characteristics make mental folding a non-rigid transformation (Taylor and Hutton, 2013; Harris et al., 2013a; Ormand et al., 2014) and potentially a more challenging representation than rigid ones (Harris et al., 2013b; Angerer and Schreiber, 2019; Hilton et al., 2022).

Mental folding allows one to transform a two-dimensional form into a three-dimensional one, while mental rotation can not practice this representation since it is a rigid mental transformation process (Atit et al., 2013; Hinze et al., 2013; Harris et al., 2013b). Mental folding also significantly contributes to STEAM success (Burte et al., 2017; Hodgkiss et al., 2018; Toub et al., 2019); indeed, a number of studies advocate that mental folding skills may be even more beneficial than mental rotation skills in supporting spatial cognitive development (Taylor and Hutton, 2013; Harris et al., 2013a; Hodgkiss et al., 2018). However, the literature lacks training studies on mental folding. Existing studies tackle training in mental folding through origami, the Japanese art of paper folding, since origami leads individuals to explore forms of various three-dimensional structures (Tenbrink and Taylor, 2015; Megahed, 2017; Wu and Sun, 2020). On the other hand, various toy designs in the market share particular affordances with the paper folding activity, and consequently, they may also improve spatial reasoning. For example, toys can aid the mental folding skill set when implemented in non-rigid assembly systems (Atit et al., 2013; Taylor and Hutton, 2013), allowing the construction of an endless number of geometries and transformation from two-dimensional forms to three-dimensional forms (Kudrowitz and Wallace, 2010; Rigo et al., 2016; Münzer et al., 2018). We suggest that these affordances must be implemented in construction toys more often (design recommendation 1). The following section will exemplify some toys that may enhance mental folding skill.

### 4.4. Toy examples for enhancing mental folding

**Figure 4:**
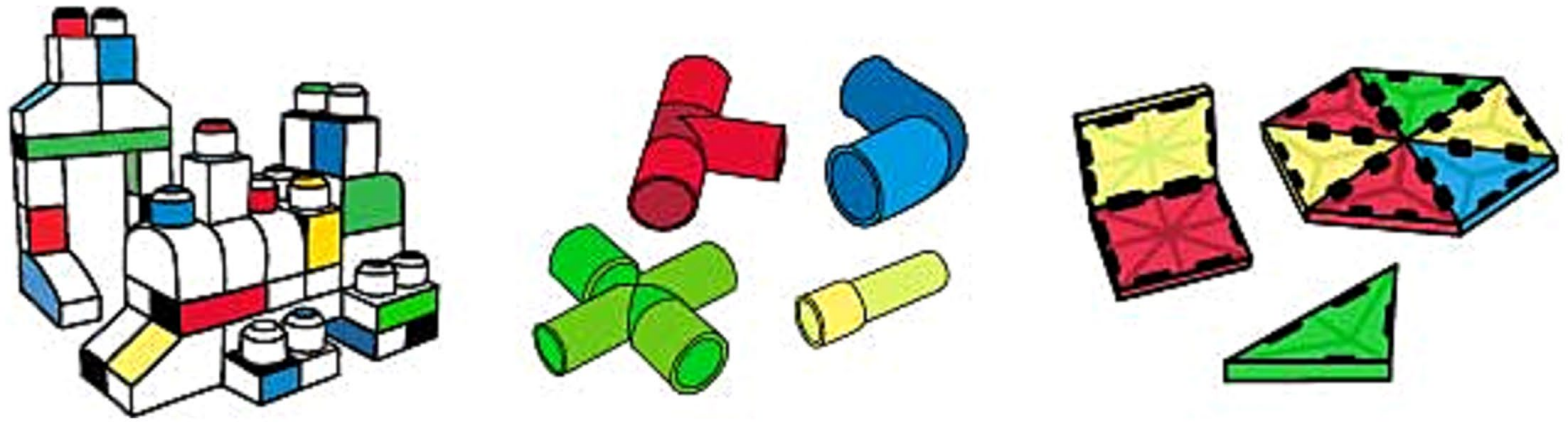
Toy examples to foster mental rotation (Mega Bloks on the left) and mental folding (middle to right; ZoZoplay & Magna Tiles).

Prototypical building blocks have several weaknesses in improving multiple aspects of spatial cognition. Many of the construction toy units in that group are inspired by the shape of a brick such as LEGO® and Mega Bloks. These toys’ three-dimensional volume of cubic geometries is divided into two-dimensional standard reference planes within vertical or horizontal axes. This causes the toys to be played with only by focusing on one surface of the object at a time (i.e., creating a tower by placing the pieces on top of each other or creating a wall by placing them side by side) (Reifel, 1984; Rode and Cucuiat, 2018; Polinsky et al., 2022). Due to the cubic form of the pieces, the construction units of LEGO^®^, Mega Bloks, etc., (see Figure 4) offer a rigid transformation during the play experience (Atit et al., 2013; Resnick and Shipley, 2013; Harris et al., 2013a), and these toys’ contribution to spatial reasoning is limited to mental rotation skill.

**Figure 5:**
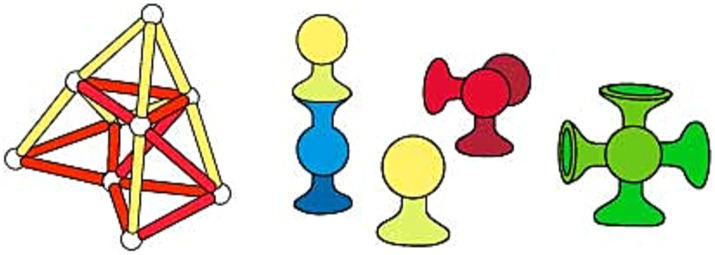
Toy examples to foster mental folding cont. (left to right; GeoMag & Squigz Fat Brain Toys).

On the other hand, practicing mental folding skills requires a non-rigid transformation during play experience by producing alternative geometries to cubicle configurations. Besides that, transforming initial two-dimensional physical properties and mental representations of objects into three-dimensional ones is necessary for improving mental folding skill (Hilton et al., 2022). Various toy designs have these features, such as ZozoPlay, Magna-Tiles, GeoMag, and Squigz Fat Brain Toys (see Figures 4, 5). Pieces of ZozoPlay are made of pipe-like modular shapes that come in different forms. Each unit has one small and one wide end to indicate the joint mechanism embedded within the design. Magna Tiles are made of flat, primitive-shaped, modular plates with magnetic fields around their edges to assemble pieces. Additionally, GeoMag consists of two main elements: spikes and balls. Spikes are short, flat bars with magnetic fields on their ends to signify where the ball can be assembled. The ball is, on its own, a magnetic ball that can be easily attached to other pieces. Furthermore, toys like Squigz Fat Brain Toys have an assembly system that holds each piece together without benefitting from the magnetic field. Squigz Fat Brain Toys utilize a vacuum to attach pieces together as the joint system. All the pieces in the previously presented toys are small, so they can be easily manipulated, carried around, and played with. In this group of toys, the play experience usually starts with constructing two-dimensional primitive closed geometries. The activity will be transformed into three-dimensional geometries by adding pieces to the z-axis with a certain angle or bending the shape from a particular edge. There are endless combinations the pieces can attach to since the joint mechanisms enable users to compose undefined shapes and geometric structures. Thanks to these non-rigid transformations, users can mentally visualize the manipulation they will apply, then transform the object from two-dimensional to three-dimensional, which may utilize mental folding capabilities in return. Lastly, since all the folding activities require a certain angle of rotation (Hilton et al., 2022), these toys may enhance both mental rotation and mental folding skills at the same time.

### 4.5. Perspective taking

Like mental folding, training of the extrinsic-dynamic elements of spatial cognition, dubbed as perspective taking skill, also requires more attention given that its social aspects receive more focus than its spatial characteristics. Indeed, in the literature, it is acknowledged that perspective taking skills can be divided into subgroups: visual perspective taking (understanding how a scene looks from another frame of reference), affective perspective taking (an individual’s ability to understand that others may feel different emotions than oneself), cognitive perspective taking (individual’s ability to reason about other people’s thoughts) (Kurdek and Rodgon, 1975; Newcombe, 1989; Yadollahi et al., 2022). Cognitive perspective taking skills form the basis of the Theory of Mind (Selman, 1980; Barnes-Holmes et al., 2004; Apperly, 2012), and affective perspective taking forms the basis of empathy (Ruby and Decety, 2004; Lamm et al., 2007; Erle and Topolinski, 2015). Among these categories, researchers focus on enhancing cognitive perspective taking skills the most. There are many interventions for cognitive perspective taking, and a few for affective perspective taking; yet, there are lack intervention studies with children that are devoted to visual perspective taking skill (Uttal et al., 2013b; Mori and Cigala, 2015; Vander Heyden et al., 2017). A recent study by Tian et al. (2021) employs a visual perspective taking training. However, this study’s initial aim is not to enhance visual perspective taking; rather, they investigate the link between the Theory of Mind and spatial skills. The spatial cognition training with construction toys supports the direction of the causal relationship such that improvement in spatial cognition leads to an improvement in the Theory of Mind performance, owing to the mediatory mechanism of perspective taking (Tian et al., 2021). A potential explanation for the shared mechanism between the Theory of Mind ability and spatial cognition can be the traditional Level 1 & Level 2 perspective taking framework proposed by Flavell (1974). In this model, two levels of visual perspective taking are defined: Level 1 refers to the understanding that other individuals may have a different line of sight and the ability to determine what others can and cannot see, while Level 2 perspective taking is the understanding that others may see things differently, and the ability to determine the positions of objects from the other’s point of view (Flavell, 1974; Kessler and Wang, 2012; Frick et al., 2014).

A classical referential communication task created by Keysar et al. (2000) also demonstrates the importance of the perspective taking skill in a social communicational setting. Researchers provided participants with a shelf with 16 slots; some slots had an item within, and some were empty. All the items are visible from the addressee’s (the participant’s) view, but some are blocked from the vision of the director (a research assistant) sitting on the other side of the shelves. The participant’s task is to rearrange the shelves with the instructions of the director. For example, there are three candles on the shelves: a small candle, a medium candle, and a big candle. However, the small candle is blocked from the director’s perspective; s/he can only see the medium-sized candle and the big candle. When the director asks the addressee to move the small candle, s/he must take the director’s perspective and determine that s/he must be referring to the medium size candle, as the smallest one is blocked from his/her view. This is a visual perspective taking task, and it also demonstrates perspective taking skill’s communicational role to establish common ground between the addressee and the director (Keysar et al., 2000; Nilsen and Fecica, 2011; Kessler and Wang, 2012).

### 4.6. Toy examples for enhancing perspective taking

Both social and spatial aspects of the play experience can be enhanced within an informal family setting by altering and referring to various configurations of the toys enabling manipulation. Yet, current toy designs usually use the brick system as the construction unit. Bricks are usually quite small, and as it is mentioned in the previous sections, their only affordance allows construction in the x and y axes (Reifel, 1984; Rigo et al., 2016; Rode and Cucuiat, 2018); consequently, much of the space exploration will be disregarded during play. This problem can be overcome by expanding the size of the units and adding joint mechanisms that afford to construct alternative geometries such as spherical ones (design recommendation 2), since large-scale units and spherical geometries enable individuals to expand their use of space and move between objects, which require exercise of the extrinsic-dynamic spatial cognition (Sas and Mohd Noor, 2009; Münzer et al., 2018; Cardillo et al., 2020).

**Figure 6:**
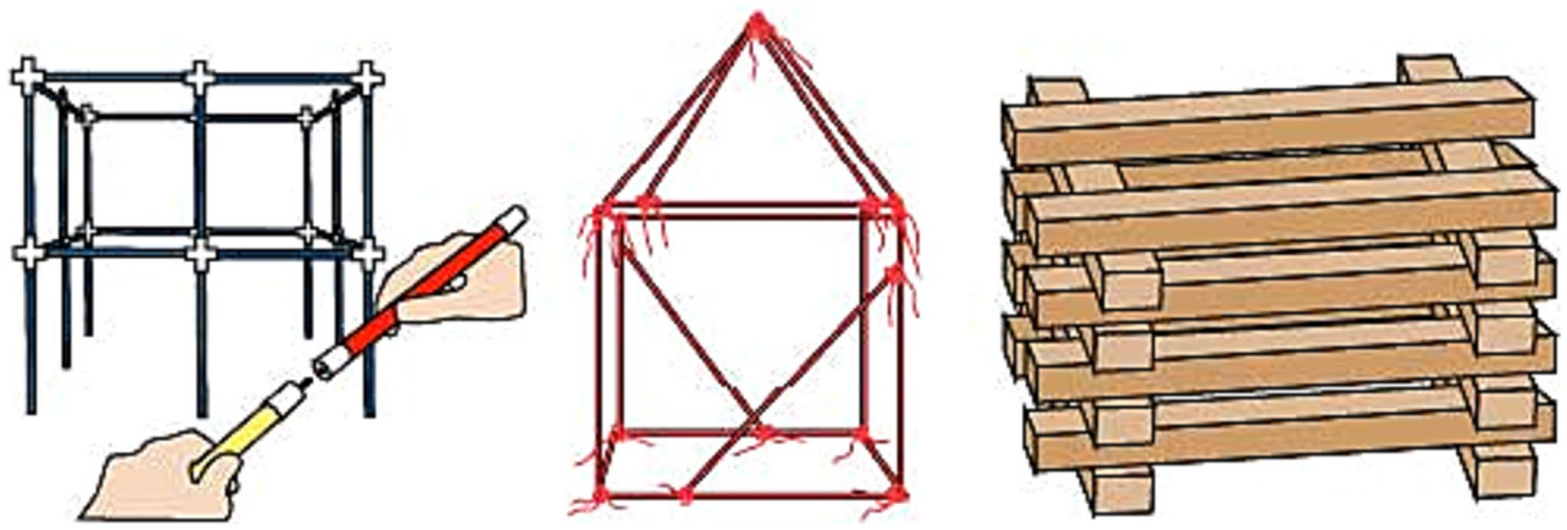
Toy examples to foster perspective taking (left to right; Strawctures, Stocks, Blockspot®).

Strawctures (Yu et al., 2022) and Stocs may provide previously mentioned affordances to practice perspective taking (see Figure 6). Strawctures consist of pipes and wheel-like stabilizers for the corners. Stocs are simply consolidated ropes that can create outline structures such as a tent or a boat by knotting. These toys provide an alternative to the linear motion field by creating different three-dimensional shapes, such as spherical geometries, owing to the assembly system of units allowing to join them in various angles and combinations. The advantage created by spherical geometries is that the toy encourages movement in a larger volume in the three-dimensional space, and the modularity of the design allows the shape to be as large as the player desires. For these reasons, it can be said that designs enabling spherical geometry may be more amenable to exercising perspective taking (design recommendation 2).

**Figure 7:**
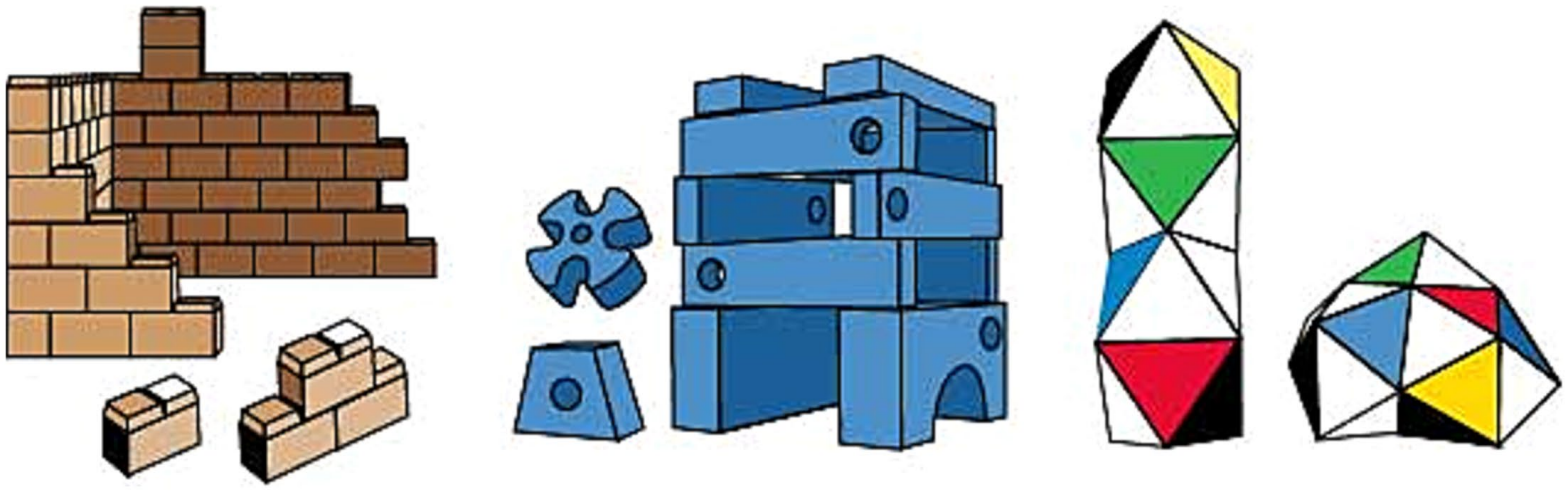
Toy examples to foster perspective taking cont (left to right; Gigi Blocks, Imagination Playground, The Toy).

Another way to enhance perspective taking skills is to establish an interaction within the larger volume by increasing the size of construction units. Blockspot^®^ is an example of a construction toy that consists of large units (see Figure 6), triggering the user to walk around the compositions and use the play space holistically (Cohen and Emmons, 2016), thus encouraging the use of extrinsic spatial cognition. Another candidate for enhancing perspective taking skill is Gigi Blocks, which consists of large cardboard blocks with tabs on the top and gaps underneath, similar to the LEGO® ‘s brick system, aside from the size (see Figure 7). These cardboard bricks can be stacked on top of each other to build real size structures. Moreover, Imagination Playground is a large-scale construction set made of foam blocks, some circular, some cubic, etc., modeled after archetypal playground elements encouraging children to build their own playground (see Figure 7). The modules can be stacked on top of each other or attached using connectors that fit into the holes in the building blocks. Since the sizes of the blocks are large, children can walk through their compositions and experience their building as a whole. Lastly, The Toy is a large-scale construction set made of fiberglass sticks and 30-inch triangle and square panels made of vinyl (see Figure 7). It can compose anything from tents to houses to tunnels (Ginoulhiac, 2013). While each toy offers different play opportunities based on its affordances, they all encourage its users to take different points of view while interacting and building with the toy, positively impacting perspective taking skills.

Mental rotation, mental folding, and perspective taking skills were presented among the physical properties of construction toys. On the other hand, these toys offer affordances beyond physicality. A construction play experience elicits verbal, gesture, and narrative interaction, which also contribute to spatial mental representations. Construction toys’ interactional affordances, which invite fruitful play interaction in spatial cognition development, will be discussed in the next section.

## 5. Features of construction toys to facilitate play experience

Designing play interactions is as important as designing the physical properties of the toys (Wooldridge and Shapka, 2012; Black et al., 2016; Yamada-Rice, 2018) (design recommendation 3). For instance, one of the major strengths of construction toys is to promote spatial talk during play sessions (Ferrara et al., 2011; Levine et al., 2012; Yang and Pan, 2021) and various toys tend to elicit spatial language in different amounts (Verdine et al., 2014). There is a link between spatial language use and improvement in mental rotation skill (Polinsky et al., 2017; Ralph et al., 2020; Turan et al., 2021). The language used during construction play enables one to create and express mental representations, and triggers spatial thinking (Casasola et al., 2020; Bower et al., 2020a; Miller-Goldwater and Simmering, 2022). Designing play experience is as important as designing the toys’ objecthood in the toy design process (Wooldridge and Shapka, 2012; Verdine et al., 2014; Black et al., 2016). Play experience entails all the communicative interactions children experience around the toy, including with their parents, teachers, peers, etc. when they play collaboratively (Healey and Mendelsohn, 2018; Ralph et al., 2020). This is also relevant for spatial play since it elicits spatial language (Ho et al., 2017; Verdine et al., 2019; Casasola et al., 2020). According to the coding scheme proposed by Cannon et al. (2007), spatial language can be captured through words describing spatial features (e.g., near, in front of, next to, tilt it down, etc.) and properties of objects (e.g., big, short, square, round, etc.). It has been shown that parents use more spatial language when playing with blocks (Ferrara et al., 2011) and puzzles (Levine et al., 2012), and when parents use more spatial language, children’s usage increases as well (Pruden et al., 2011; Kısa et al., 2018; Clingan-Siverly et al., 2021).

Enabling guided play scenarios, where spatial language is encouraged, is an opportunity for implementing linguistic input into play. In guided play, adults focus the child’s interest on the learning objectives by using verbal scaffolding, asking open-ended questions, posing problems, thinking out loud, praising and encouraging discoveries made by the child (Fisher et al., 2013; Weisberg et al., 2013; Cohen and Emmons, 2016). It has been demonstrated in multiple studies that children benefit from guided play more than they do from free play or didactic play in terms of learning new skills, including mental rotation (Fisher et al., 2013; Ramani et al., 2014; Borriello and Liben, 2017).

Furthermore, guided play with construction toys can be an engaging way of improving spatial cognition for adults and children simultaneously. However, to our knowledge, no studies investigated the mutual benefits of guided play for adults and children. Studies assert that spatial skill malleability does not exclude adults (Uttal et al., 2013a; Cherney et al., 2014; Kornkasem and Black, 2015). Adults may also benefit from interacting with the right instrument designed to enhance spatial skills; however, the literature lacks an engaging way to develop adult spatial cognition (Newcombe, 2017). Guided building play can improve adults’ spatial skills, as it helps to improve children’s, since adults also enjoy interacting with construction toys (Ginoulhiac, 2013; Toub et al., 2019). Previous literature implies that spatial gains may be simultaneous for parents and children dyads engaging in spatial play, no particular study sheds light on this subject.

In terms of the content of the guided play, presenting a training stimulus in combination with either realistic or fantastic but especially with fantastic narrative context is found to be beneficial for learning in many domains, for instance, word learning (Weisberg et al., 2015). The same is also valid for spatial cognition, such that the benefit a child gains from the language produced during play can be enhanced by adding a narrative component (Rohlfing and Nachtigäller, 2016), since the narrative may motivate the children and make the play experience more engaging (Casey et al., 2008). In addition to helping with the learning process, narrative input is known to help retain what is learned (Bower and Clark, 1969; Graesser et al., 1980). Thus, implementing thematic elements such as animals or human characters into the construction toy can lead children to create stories, engage more enthusiastically with the enacted story world, and interact more in a spatial manner.

However, it must be noted that the thematic pieces should be supplementary material rather than the main focus as they may distract the child and jeopardize the spatial characteristics of the play activity (Stanton and Weisberg, 1996; Wellhousen and Kieff, 2001; Tunks, 2009). To compose an architectural setting once and create stories inside the structure, such as a doll house, is not an efficient way of practicing spatial skills because building and rebuilding multiple times is the key parameter for practicing spatial representations (Jirout and Newcombe, 2015; Fanning, 2018; Rode and Cucuiat, 2018). The aforementioned disadvantageous thematic elements can be observed in the themed sets based on objects, vehicles, and buildings from media such as Star Wars, Harry Potter, Lord of the Rings, etc. These product lines act more as collection items than construction toys (Wolf, 2014; Fanning, 2018). Once individuals get these sets, they follow the instructions, complete the suggested composition and rarely pull it apart again. In this way, the construction pieces become display material and lose their ability to promote spatial thinking when built only once. Another side effect of the thematic product lines is that the benefit of stimulating creativity would be lost when predetermined instructions are followed instead of free construction (Moreau and Engeset, 2016; Fulcher and Hayes, 2017; Rode and Cucuiat, 2018).

Although this issue is mostly considered in relation to children’s spatial learning and play experience, it is worth noting that adults’ spatial gains also suffer from construction toys that are overwhelmingly themed. Construction materials targeting adults are almost exclusively of this sort and lack the assembling and reassembling aspects that promote spatial learning. As previously mentioned, adults can benefit from spatial training either by themselves or engaging in guided play (Newcombe et al., 2013; Cherney et al., 2014; Kornkasem and Black, 2015). Through a convenient design that engages both the adult and the child, construction toys can eliminate the age gap in the market and bring adults and children together in a way that creates an opportunity for mutual benefit gathered from a single training tool.

Gesture production is another scaffolding tool children can benefit from while playing with construction toys, especially if they are designed to encourage both the child and the adult to produce gestures (Kısa et al., 2018; Bower et al., 2020b; Clingan-Siverly et al., 2021). Both gesture production and observing someone while gesturing are valuable for fostering spatial reasoning (Ehrlich et al., 2006; Chu and Kita, 2011; Toub et al., 2019). Besides, Goldin-Meadow et al. (2012) revealed that gesture production is more effective for mental rotation improvement than observing someone while gesturing. Gestures’ contribution to mental rotation skill is rooted in the communicating visuospatial modality (Baykal et al., 2018; Yang et al., 2020). For instance, gestures can represent objects, directions, and orientations (Alibali, 2005; Galati et al., 2017; Karadöller et al., 2021); they essentially allow spatial language to be converted to physical expressions. Indeed, gestures are situated in the middle of the visual and verbal expression styles (Newcombe et al., 2013). In an empirical study, Stieff et al. (2016) demonstrated that gestures foster STEAM performance by converting the imagined movement of mental representations into a concrete movement in the physical space. In this way, gestures provide solutions for the spatial visualization challenges, which can be encountered in STEAM tasks (Chu and Kita, 2011; Stieff et al., 2016). It is also found that worse performers in the traditional paper and pencil mental rotation task tend to gesture more to convey static information in comparison to those who performed better in mental rotation (Göksun et al., 2013), demonstrating that individuals strive to resolve a cognitively demanding spatial task for them through the aid of gestures and overcome the challenge. Using gestures during spatial activities can facilitate spatial skills; however, few studies investigate the role of gesture input in spatial reasoning (Yang et al., 2020; Clingan-Siverly et al., 2021).

To sum up, the interactions that engage adults with their children can provide opportunities for both parties to benefit since studies demonstrate that adult spatial cognition is also malleable (Uttal et al., 2013a; Cherney et al., 2014; Kornkasem and Black, 2015) and adults also enjoy playing with construction toys (Ginoulhiac, 2013; Toub et al., 2019), although there is no inclusive, enjoyable intervention for their spatial skills (Newcombe, 2017). Therefore, concept designs that invite children and adults to play and benefit together must be produced (design recommendation 4). To date, no research has investigated the simultaneous cognitive benefits of construction play for adults and children. Still, there are studies showing adults scaffold children’s spatial development (Vygotsky, 1978; Trawick-Smith, 1998) by using narratives (Casey et al., 2008), spatial language (Ferrara et al., 2011; Pruden et al., 2011; Cohen and Emmons, 2016), and gestures (Chu and Kita, 2011; Kısa et al., 2018; Clingan-Siverly et al., 2021). Features of language, narrative, and gesture input must be incorporated into the play experiences (Verdine et al., 2014) to facilitate at-home STEAM development (design recommendation 5). Furthermore, affordances provided by the construction toys must be varied with the choice of more abstract units to build unlimited combinations (Reifel, 1984; Ginoulhiac, 2013; Trawick-Smith et al., 2014) as opposed to contemporary licensed thematic sets, in which individuals consistently replicate the forms of popular movie settings (Wolf, 2014; Fanning, 2018) and create display materials in which narrative features shadow the construction play (design recommendation 6).

## 6. Discussion and conclusion

Access to quality STEAM education starting from the preschool period is known to be a predictor for future academic success (Gözüm et al., 2022). Thus, play interactions are fruitful investments for joyfully and effectively increasing STEAM success in an informal context, in view of strong evidence for the link between well-developed spatial cognition and achievement in the STEAM-related fields. A multitude of studies demonstrate that playing with construction toys enhances spatial reasoning; thus, making construction toys an accessible tool contributes to informal STEAM development. Although certain aspects of spatial skills in relation to construction toys have been already investigated, this paper pointed out several gaps in the research area. To bridge this gap, it revisited developmental psychology and design literature, presented existing discussions in the developmental psychology field and some construction toy examples available in the toy market and design studies through a benchmark. In the end, six recommendations were identified to provide guidance about what could be done further to support both toy design and developmental psychology by strengthening the link between the two fields.

One of the main takeaways of this paper is that developmental psychology and design fields should collaborate more to design toys that contribute to informal STEAM development in children. Design researchers have an important role in this regard as they can act as the mediators of theoretical knowledge derived from developmental psychology, who turn empirical knowledge into actionable design guidelines for design practitioners. The first research question in this paper was which findings from developmental psychology had not yet been applied to toy design. In search of an answer, we created a benchmark for construction toys’ potential contributions to spatial skills, which is prepared in accordance with spatial affordances of over fifty toys from the market and design research studies (see Table 2 for the spatial skill contribution of the toys, and Appendix A for the toys’ relevant affordances). A thorough review of these toys demonstrated that existing construction toys focus more on supporting mental rotation skill, while very few address mental folding and perspective taking skills. This seems to be a missed opportunity for design, indicating a need to integrate design features that can support various spatial cognition skills (i.e., mental folding and/or perspective taking in addition to mental rotation). The benchmark showed that the main problem in this literature is the need to investigate other construction units in addition to the typical brick system in spatial toys. Accordingly, there are very few toy options that can foster perspective taking and all three skills together, although various toy designs may contribute to different spatial skills. Studies conducted with these toys are limited to small-scale user studies. Empirical methodology with larger sample sizes comparing different toys’ affordances as stimuli can provide evidence regarding the positive impact of toys on the three spatial skills (i.e., mental rotation, mental folding, and perspective taking).

**Table 2:** Benchmark for the construction toys’ potential contribution to spatial skills.

	Mental rotation	Mental rotation and mental folding	Mental rotation and perspective taking	Perspective taking	Mental rotation and mental folding and perspective taking
	LEGO (Wolfgang et al., 2003; Wolf, 2014)	Brainflakes	Rigamajig	Tommy Blocks (Rigo et al., 2016)	Zometool
	Mega Bloks	Learning Resources Gears! Gears! Gears!	Imagination Playground (Ginoulhiac, 2013)	Sifteo (Geurts et al., 2014)	Polydron
	Unit Blocks	Squigz Fat Brain Toys	gigi Blocks	Co-gnito (Panagiotidou et al., 2022)	MagnaTiles (Ralph et al., 2020)
	Montessori Wooden Blocks (Baykal et al., 2018)	Topobo (Raffle et al., 2004; Parkes et al., 2008)	Habitadule		GeoMag
	KÜP-TAK	Posey (Weller et al., 2008)			Strawctures (Yu et al., 2022)
	Lincoln Logs (Ginoulhiac, 2013)	Kinematics (Oschuetz et al., 2010)			Stocs (Vander Heyden et al., 2017)
	Jeujura Wooden Construction Toy	ZoZoplay			K'Nex
	Bristle block	Vkoizzi			Geemo (Ginoulhiac, 2013)
	Learning Resources City Engineering	Plus-Plus			DIY Model Doll House
	Fischertechnik	Tinkertoy (Baykal et al., 2018)			Marble Maze (Vander Heyden et al., 2017)
	Kunmark (drill toy)	Toyi (Agirbas et al., 2022)			The Toy (Ginoulhiac, 2013)
	Pontiki	Clixo			
	Jigsaw Puzzle (Levine et al., 2012)	Wikki Stix (Baykal et al., 2018)			
	Tangram (Baykal et al., 2018)	Wacky Tracks			
	Katamino	Pop Tubes			
	Q.Bitz	Speks Flex			
	Boda Blocks (Buechley and Eisenberg, 2007)	Legoon (Yang and Druga, 2019)			
	AlgoBrix				
	Pixio				
Total:	19	17	4	3	11

The second research question was how to present the knowledge obtained regarding the first question to designers in a feasible way while effectively closing the gap between the two disciplines. In order to do so, the findings from theoretical foundations of developmental psychology were combined with the design features of the construction toys to demonstrate market tendencies for enhancing spatial skills (see Table 2). Additionally, we produced six design recommendations that designers can refer to while developing new toys (see Table 3). These recommendations include some key points for the toy design as well as designing the play experiences, since designing a play interaction is as important as the physical features of the construction toys. If training spatial skills in young children will be achieved, their adult partners (parents, teachers, etc.) must be encouraged to produce the necessary spatial input (e.g., spatial talk or gesture) for the emergence of spatial play. Another potential contribution of engaging adults in spatial play is that they may also benefit from this interaction in the form of spatial skills development. Thus, the interests of different personas (i.e., adults and children) should be considered. In line with these insights and background literature, the design recommendations were prepared to inspire designers and fill the gaps in this area of literature.

**Table 3:** Recommendations for toy designers.

Include mental folding components in toys, as mental folding also has value for STEAM success (Harris et al., 2013a; Resnick and Shipley, 2013; Taylor and Hutton, 2013; Burte et al., 2017; Hodgkiss et al., 2018; Toub et al., 2019) Design large-scale or spherical toys to facilitate perspective taking skill, as perspective taking is related to both spatial and social skills (Sas and Mohd Noor, 2009; Shelton et al., 2012; Tarampi et al., 2016; Vander Heyden et al., 2017; Cardillo et al., 2020; Tian et al., 2021) Consider the entire user experience playing during the design process rather than focusing only on the physical properties of the toys (Wooldridge and Shapka, 2012; Weisberg et al., 2013; Black et al., 2016; Healey and Mendelsohn, 2018; Yamada-Rice, 2018; Verdine et al., 2019) Embrace multiple personas, such as parents and children playing together, so both the child and the adult can benefit from enhancing spatial skills (Lin, 2010; Uttal et al., 2013a; Ginoulhiac, 2013; Cherney et al., 2014; Kornkasem and Black, 2015; Borriello and Liben, 2017) Design toys that will elicit spatial language, narrative, and gesture use as much as possible (Casey et al., 2008; Ferrara et al., 2011; Pruden et al., 2011; Verdine et al., 2014; Stieff et al., 2016; Clingan-Siverly et al., 2021) Avoid using themed products that may limit imagination. The narrative must be a complementary feature without shadowing the construction play. Provide users with abstract designs to stimulate symbolic thinking and allow different configurations as an alternative to typical brick systems (Reifel, 1984; Ginoulhiac, 2013; Trawick-Smith et al., 2014; Wolf, 2014; Rigo et al., 2016; Fanning, 2018)

The final research question was what are the responsibilities that developmental psychologists had in bridging the gap between their field and toy design. On the side of developmental psychology, there is a call for more research of construction toy designs that include different affordances rather than focusing solely on typical brick-shaped units (see Appendix A). Doing so would potentially provide empirical evidence of the expected benefits of a wider range of construction toys to mental folding and perspective taking skills, skills that are overlooked, in addition to the well-studied mental rotation skills. The benchmark provided in Table 2 attempts to give a glimpse of the full picture in the construction toy market based on their potential contribution to mental rotation, mental folding and perspective taking skills. However, it should be noted that it is hypothetical to claim whether the affordances of those toys satisfy their matched spatial skills due to the lack of empirical research conducted on such toy designs. Hence, it would be beneficial for future studies to investigate the above-mentioned connections.

Overall, this review aimed to point out that there is a lack of collaboration between developmental psychology and toy design fields. Developmental psychology studies are mostly executed with a limited variety of toys (i.e., block-type construction toys) and are focused on the mental rotation skill. Alternative toy designs are not considered while facilitating spatial development, although their affordances may contribute to different aspects of spatial cognition (i.e., mental folding and perspective taking). On the other hand, toy design research barely considers theoretical frameworks from developmental psychology, or the empirical backgrounds of existing products in the market are not always clear. Through revealing these issues and investigating developmental psychology and toy design with a lens of spatial cognition, this paper initiates a dialog between these fields to foster informal STEAM development.

## Author contributions

Çİ, ME, DK, AC, TG, and AK designed and conceptualized the study. Çİ, ME, and DK reviewed the literature and wrote sections. ME and DK conducted market research, drew visuals, and prepared tables. Çİ and DK wrote the first draft of the manuscript. AC, TG, and AK read, revised, and provided feedback for the manuscript. All authors contributed to the article and approved the submitted version.

## Conflict of interest

The authors declare that the research was conducted in the absence of any commercial or financial relationships that could be construed as a potential conflict of interest.

## Publisher’s note

All claims expressed in this article are solely those of the authors and do not necessarily represent those of their affiliated organizations, or those of the publisher, the editors and the reviewers. Any product that may be evaluated in this article, or claim that may be made by its manufacturer, is not guaranteed or endorsed by the publisher.
