# Supplementary material for: Malleability of spatial skills: bridging developmental psychology and toy design for joyful STEAM development
Source: Front Psychol. 2023 Sep 12;14:1137003. doi: 10.3389/fpsyg.2023.1137003 (PMC10523793; doi:10.3389/fpsyg.2023.1137003)
Supplement: Supplementary file 1 [file Data_Sheet_1.docx]

**Appendix A: Supplementary Materials**

**Benchmark of the Construction Toys’ Affordances**

| name of the toy | affordances of the toy |
| --- | --- |
| LEGO | Small brick-like shapes come together by the tabs on the top surface and gaps underneath to build various structures. Because of the size of the bricks, they are easily manipulated by hand. |
| Mega Bloks | Have similar affordances to LEGO, except that the scale of each modular shape is bigger in Mega Bloks. |
| Unit Blocks | Are composed of different primitive shapes. They can be easily manipulated by hand, thanks to their scale. The flat surfaces on each side signify that each modular shape can be stacked on the other. There is no joint system that can hold pieces together. |
| Montessori Wooden Blocks | Are primitive-shaped modular blocks. Their scale allows them to be easily played by hand. Each modular form has at least two flat surfaces, enabling blocks to be placed on top of each other. Some blocks' edges have various endings; some are round and some are pointy. |
| KÜP-TAK | The toy is made of cube-like modular blocks. Each cube has square dents on each surface to signify the area where cubes should be interlocked together to build the structure. These small cubes indicate that they can be carried out and modified easily. |
| Lincoln Logs | Pieces of the toy are square-notched miniature lightweight logs that could be put side by side or on top of one another to build structures. The pieces are small; therefore, they can be manipulated by hand easily. The round corners make it harder to stay balanced without support. |
| Jeujura Wooden Construction Toy | The toy comprises rounded square-notched lightweight logs, flat construction pieces, and miniature lifestyle figures like windows and door frames. The logs have gaps, the same size as the logs' width, on each side to signify the joint areas. The remaining elements of the toy assume that the toy is made to build houses. |
| [Bristle block](https://www.amazon.com.tr/Bristle-Blocks-Kavanoz-56-Par%C3%A7a/dp/B004Z0VYYM/ref=sr_1_1?adgrpid=120214863099&gclid=Cj0KCQjwsrWZBhC4ARIsAGGUJurytijOH_wcC_zMGM0oq_9JHcsrM_W4G82HthH_-kptWw25QUslc58aAmlLEALw_wcB&hvadid=599795447164&hvdev=c&hvlocphy=1012782&hvnetw=g&hvqmt=e&hvrand=4402591798387316194&hvtargid=kwd-298894367951&hydadcr=18743_2268435&keywords=bristle+blocks&qid=1663947143&sr=8-1) | The set is composed of primitive-shaped modular pieces with rounded-edged spikes to signify the areas where these modules should come together. These modules are thick, allowing them to be comfortably held in hand. |
| [brainflakes](https://brainflakes.com/collections/building-instructions-and-task-cards#ball) | The toy comprises one element: a disc with holes on its end. The holes signify how the structure should be made by interlocking them. Discs are small, which indicates that they can be easily put together by hand. |
| [Plus-Plus](https://www.amazon.com.tr/Oyuncak-Plus-Plus/s?rh=n%3A12467126031%2Cp_4%3APlus-Plus) | The toy pieces are 3D puzzle-like shapes that can be placed on a base provided within the set or put together on any surface. The holes within each module signify where each should relate to one other. The pieces are small; therefore, they can be easily maneuvered. |
| [Learning Resources Gears! Gears! Gears!](https://www.learningresources.com/shop/category/engineering-building-sets) | Affordances of Learning Resources Gears! Gears! Gears! are quite similar to Plus-Plus's. The main difference is the motion that can be added to Gears, Gears, Gears. Since each element is placed and used as a disk, the cogs of the disk can be rotated when the key is used to turn them. The arrangement of each piece allows the understanding of modular systems. |
| [Learning Resources City Engineering](https://www.amazon.com/Learning-Resources-Engineering-Design-Building/dp/B0728L78KC/ref=sr_1_41?dchild=1&qid=1595601150&refinements=p_n_age_range%3A165890011%7C165936011&rnid=166269011&s=toys-and-games-intl-ship&sr=1-41&th=1) | The toy is a miniature replica of a real-life construction site. The main pieces of the toys are flat flooring and column-like stabilizers. The bulges on top of the flooring indicate where the columns should be attached to build the structure. There are additional pieces to support the balance of the structure, like connecting bulges that can be placed on top of the columns to connect them. All pieces are small so that they can be easily played and manipulated. |
| [Squigz Fat Brain Toys](https://www.fatbraintoys.com/toy_companies/fat_brain_toy_co/squigz.cfm) | The pieces of the set are the same in terms of function, yet come in different forms. Despite the changes in the form and size, each piece has one balloon-like shape in the middle while having two concave finishes that allow air to be stacked between the piece and the surface. The balloon-like shape allows pieces to be grabbed easily and pushed/pulled when necessary. Each piece is small; therefore, they can be played with and mismatched easily with one another. |
| [Fischertechnik](https://en.wikipedia.org/wiki/Fischertechnik) | The toy is made of 3D modular shapes. Each shape has a certain gap and bulges to signify the areas they can be put together. Mechanic elements can be implemented within the toy's structure to allow movement control. |
| [Kunmark](https://www.amazon.ca/dp/B07NZPFV9J/ref=sspa_dk_detail_2?psc=1&pd_rd_i=B07NZPFV9J&pd_rd_w=452uz&content-id=amzn1.sym.c7dca932-da6a-44fc-af09-cc68d2449b34&pf_rd_p=c7dca932-da6a-44fc-af09-cc68d2449b34&pf_rd_r=JBAGCGN4EMAQ2RCT0RJX&pd_rd_wg=IBCkc&pd_rd_r=00978d85-3d29-47b2-a6f1-ee398e6e2169&s=electronics&sp_csd=d2lkZ2V0TmFtZT1zcF9kZXRhaWw&smid=A241RP4T89RNDF) | The toy comprises flat modular shapes (squares, triangles, and circles) with multiple circular holes and toy tools to join these shapes: a screwdriver, a drill, a wrench, and screws. The units are combined by aligning the holes on separate shapes and screwing them together using the tools. The shapes can be rotated on a single plane, and since the holes need to be aligned to be joined, the flat shapes only allow for a 2D play; any combination of units remains flat as well. |
| [Pontiki](https://en.wikipedia.org/wiki/Pontiki) | Is a small-scale construction toy that focuses on character design. It consists of body parts, and small parts are the eyes and limbs. Body parts can be cubes, cylinders, cones, and egg shapes. The smaller parts fit the holes in the body parts. |
| Jigsaw [Puzzle](https://www.trendyol.com/puzzle-x-c1155?tyutm_source=google&tyutm_medium=cpc&tyutm_campaign=x:home_y:culture_z:hobi_t:dsa_brand-category_gl_s_&gclid=Cj0KCQjwsrWZBhC4ARIsAGGUJuokw4Y7jiUnGx-7CmARNpLCYXnd8aBaSjtUai5RpU5zSd7RUKI01xkaAvNEEALw_wcB&gclsrc=aw.ds) | A set of irregularly shaped pieces with gaps and protrusions that interlock. Each of these pieces has a designated place, and when all pieces are in the right place and rotated precisely, the mosaic of the puzzle pieces displays a meaningful image. |
| [Tangram](https://tr.wikipedia.org/wiki/Tangram) | A small-scale 2D puzzle consists of seven flat polygons: 5 triangles of varying sizes, a square, and a parallelogram. The polygons are often used to represent objects, animals, etc., in a minimalist way, using all seven pieces. |
| [Katamino](https://www.mucitpanda.com/gigamic-katamino-6-99-yas) | A puzzle game like Tetris and Tangram. Made up of 12 wooden pentominoes and a wooden board, they fit perfectly. The aim is to place them into the board without them overlapping or creating some predetermined shapes. The shapes have a 2 cm thickness, but the gameplay is two-dimensional. |
| [Q.Bitz](https://www.mucitpanda.com/mindware-q-bitz-junior) | A puzzle game consisting of cubes and an 8x8 cm playing board accommodates 16 cubes. Each set of 16 cubes has different patterns on each side. To begin playing, the player picks a random card and tries to recreate the pattern it shows using the patterns on the different faces of the cubes. Harder and easier versions of this game exist, such as Q-Bitz junior, which has 4 cubes instead of 16. 2D pattern-making toy. |
| [Boda Blocks](https://dl.acm.org/doi/10.5555/1599600.1599618) (Buechley and Eisenberg, 2007) | Boda Blocks are an experimental TUI made up of 16 cubes that light up to be green or blue, and that can be arranged in different configurations. Some connectors can be attached to any of the six sides of a cube and can be used to attach the cubes to one another. Only one connector can be attached to each surface. The software accompanying the blocks programs the cubes to display various dynamic 3D light and color patterns. |
| [AlgoBrix](https://www.algobrix.com/) | Similar affordances to LEGO with similar-sized units, LEGO pieces are compatible. Additionally, AlgoBrix allows the constructions to turn into robots. The robot can be coded to perform various actions using their function and parameter blocks with the same gaps as LEGO, placed on a plate with accompanying tabs. It is a construction toy and a TUI. |
| [Pixio](https://pixio.site/) | Comprises 8x8x8mm magnetic cubes that can be attached to each other on all sides. Allows constructing 2D and 3D shapes, animals, abstract creations, etc. The small size makes the units easy to manipulate by hand. Reminiscent of Minecraft. |
| [Topobo](https://topobo.com/) (Parkes, Raffle, and Ishii, 2008; Raffle, Parkes, and Ishii, 2004) | A system construction toy that is made up of “active” and “passive” parts. Passives are static pieces that are used as building blocks. They have bone-like organic shapes and are used to construct 3D biomorphic forms. These limb-like structures can be attached to each other or active from their two ends and a notch in their middle. Active units are motorized and can be connected to each other. They have kinetic memory and replicate movement, allowing the form to move around. |
| [Posey](https://dl.acm.org/doi/10.1145/1347390.1347402) (Weller, Do, and Gross, 2008) | Like the biomorphic form of Topobo. The organic 3D shapes are joined using a ball-socket system. Connection pieces called “struts” have balls on two sides that fit the corresponding sockets in the remaining shapes. Depending on the number of sockets they have, these pieces are called “one-hub”, “two-hub,” etc. The different configurations can look like skeletons (called puppets) or molecules. Once constructed, the skeleton becomes a tangible interface and can be used to animate the puppet on screen using the accompanying software. |
| [Kinematics](https://doi.org/10.1145/1709886.1709938) (Oschuetz, Wessolek, and Sattler, 2010) | Construction toys consist of active and passive parts. The system and the constructed skeletons are like Posey and Topobo, but the shape of the active and passive parts is geometric. The units are joined with a plug-and-socket system. The active parts move in various ways and don’t require programming. Some blocks change shapes or rotate. Others provide electric power and kinetic energy. Endless combinations can create biomorphic and abstract forms that move in various ways. |
| [Tommy Blocks](https://doi.org/10.1145/2839462.2872959) (Rigo et al., 2016) | Are made of magnetic low-poly pieces. The pieces are attached to one another by their magnets. The shapes’ triangular faces don’t allow for cubic forms to be created, encouraging the creation of more atypical forms. There is a “main block” named Tommy. Tommy has a speaker and can imitate animal sounds, and another component of the toy is to build Tommy into the animal it speaks as. |
| [ZoZoplay](https://www.amazon.com/ZoZoplay-Learning-Educational-Building-Multicolor/dp/B07MPFY87L/ref=sxin_14_pa_sp_search_thematic_sspa?content-id=amzn1.sym.38deba80-70f1-44b5-9ee5-a7944caadcc1%3Aamzn1.sym.38deba80-70f1-44b5-9ee5-a7944caadcc1&cv_ct_cx=connects%2Bbuilding%2Btoys&keywords=connects%2Bbuilding%2Btoys&pd_rd_i=B07MPFY87L&pd_rd_r=047c3b1d-bb01-4620-9523-c369cb227943&pd_rd_w=Se1mi&pd_rd_wg=TyzTb&pf_rd_p=38deba80-70f1-44b5-9ee5-a7944caadcc1&pf_rd_r=W9CE16NWPQB53BXW2VSC&qid=1663943783&sr=1-3-a73d1c8c-2fd2-4f19-aa41-2df022bcb241-spons&th=1) | Pieces of the toy are made of pipe-like modular shapes, which come in different forms. Each form has one small and wide end to indicate the joint mechanism embedded within the design. The pieces are small so they can be easily manipulated, carried around, and played with. |
| [Vkoizzi](https://www.amazon.com/Vkoizzi-Sensory-Building-Blocks-Autistic/dp/B09P5JT7MY) | The toy consists of only one modular shape to construct the play. This modular shape is square with dents and tabs that initiate where parts should come together. Due to the form of the modular shape, it can only allow cubic forms to be created. |
| [Zometool](https://en.wikipedia.org/wiki/Zome#Construction_set) | Zoometool consists of two main elements: spikes and balls. Spikes are short, flat bars with tabs on their ends to signify the area to insert while combining with the ball. The ball has many holes on its surface to provide variations to bring these two elements together. |
| [Polydron](https://www.tts-group.co.uk/crystal-polydron-classroom-set-184pcs/1017633.html?cgid=Early_Years-Construction-Construction_Sets) | There are five different pieces that comprise the toy. These are triangles, squares, pentagons, and hexagons which are all flat, thin, filled slices, while the last piece is a hexagon with a flat, empty form. The last piece can be easily described as a hexagon frame. Each shape has dents and tabs to indicate where pieces come together to create the structure. Different shapes of the pieces allow the output and joining forms to vary. All the pieces are small and can be easily manipulated and played with. |
| [MagnaTiles](https://en.wikipedia.org/wiki/Magna-Tiles) | The toy is made of flat, primitive-shaped, modular layers with magnetic fields around its edges to bring the pieces together. Each piece is small, which allows the structure to be built easily by hand. |
| [GeoMag](https://en.wikipedia.org/wiki/Geomag) | Is like Zometool in terms of the shapes it is composed of. Instead of dents and tabs on each element, the pieces are structured using the magnetic field. Each stripe has magnetic tiles inside its form, while the balls are magnets. All its pieces are small, indicating that they can be easily placed, replaced, and played with. |
| [Strawctures](https://doi.org/10.1145/3490149.3501322) (Yu et al., 2022) | The toy is constructed upon two main parts the pipes, wheel-like stabilizers, and a joint mechanism. The mechanism mimics two plus signs intertwined, while the pipes look like straws. The pipes are filled with electronic wiring to allow electricity to transfer, and the ends signify how joint mechanisms should be used to connect pieces together. These elements' affordances allow the toy to be built in any size, small or big. They are easily manageable and can easily structure by hand. |
| [K'Nex](https://en.wikipedia.org/wiki/K%27Nex) | The set includes only two elements, the joint mechanism and the rods. The joint mechanism comes with some differences in terms of its shape. It looks like a fan set to different angles, allowing variations in the toy's structure. Each mechanism has holes to signify where rods should be placed to create structure. |
| [Tinkertoy](https://en.wikipedia.org/wiki/Tinkertoy) | This set includes spools, sticks, wheels, caps, couplings, pulleys, and short pointed sticks. Apart from sticks, all the elements have holes to signify where sticks should be implemented to build the structure. All pieces are small; they can be easily put together and separated and carried around easily. |
| [Toyi](https://toyi.io/tr/) (Agirbas et al., 2022) | The set has 8 pieces: circular elastic bands, sticks, wheels, and figures that mimic the human body, such as eyes, joints, hands, and feet. Dents in each piece signify the areas where modules can come together to create various structures. The elastic bands offer malleability while using the material, providing opportunities to interact with other objects outside the set. |
| [Clixo](https://clixo.com/) | Elements of the toy set are flat, flexible magnets that come in various shapes. Magnets allow pieces to stick to each other quickly and snap into a form easily. The ease in forming different shapes by folding and bending these materials comes from the material used. Each piece is small and can easily fit into hands. Therefore, one can take them wherever they go and play. |
| [Wikki Stix](https://www.wikkistix.com/) | 8-inch colorful sticks that can bend and stir to each other and other flat surfaces to make 2D & 3D shapes, patterns, and figures. |
| [Wacky Tracks](https://www.amazon.com/dp/B08YY7JLW6/ref=sspa_dk_detail_3?pd_rd_i=B08YY7JLW6&pd_rd_w=A72Lc&content-id=amzn1.sym.3481f441-61ac-4028-9c1a-7f9ce8ec50c5&pf_rd_p=3481f441-61ac-4028-9c1a-7f9ce8ec50c5&pf_rd_r=7QS7E6678YSZNXCFP7J8&pd_rd_wg=3MDcI&pd_rd_r=929e210a-0140-4adc-88d5-a945d3c9b8f0&s=toys-and-games&sp_csd=d2lkZ2V0TmFtZT1zcF9kZXRhaWxfdGhlbWF0aWM&spLa=ZW5jcnlwdGVkUXVhbGlmaWVyPUFPTDY5MElDVFpCSTAmZW5jcnlwdGVkSWQ9QTA5NTU1MjVVUExGR1RJTExVN08mZW5jcnlwdGVkQWRJZD1BMDcwODA1MTJPNjlKWFZVQ1cyVUMmd2lkZ2V0TmFtZT1zcF9kZXRhaWxfdGhlbWF0aWMmYWN0aW9uPWNsaWNrUmVkaXJlY3QmZG9Ob3RMb2dDbGljaz10cnVl&th=1) | A chain made up of 1 cm plastic pieces that snap together. The chain can bend from the connection points by pivoting the plastic units. It can be used to make 2D and 3D shapes; however, the 3D shapes are very limited due to the lack of flexibility of the connections. |
| [Pop Tubes](https://www.amazon.com.tr/s?k=pop+tubes&language=tr_TR&adgrpid=120385203535&gclid=Cj0KCQjwsrWZBhC4ARIsAGGUJuouLZX7crncR-7cUUehDWi86lVOMekq9a6nGEQduVPcTf0n6B57o94aAolIEALw_wcB&hvadid=599730439159&hvdev=c&hvlocphy=1012782&hvnetw=g&hvqmt=e&hvrand=2333365053642323883&hvtargid=kwd-304695506542&hydadcr=2931_2306005&tag=trtxtgostdde-21&ref=pd_sl_498a01u7r8_e) | An accordion tube that is 9 inches when compressed. It can be pulled, twisted, or bent. Only the ends can be attached to create a long tube, circle, etc. Some larger versions exist as well. |
| [Geemo](https://casholman.com/geemo/) | The building units of this toy are large and abstract. They appear to be limb-like, some with two arms, some with three. Their limbs are magnetic and flexible. They can be bent and repel or grab each other, allowing for the construction of equally abstract and bizarre structures. |
| [Speks Flex](https://www.amazon.com/Speks-Fleks-Silicone-6-Piece-Building/dp/B0B4F667VJ?th=1) | Flexible silicon X’s with magnets on the 4 ends, roughly 4 inches in size. They snap together; multiple x’s can be combined to make different abstract shapes. Like Geemo, but small-scaled. |
| [Legoon](https://doi.org/10.1145/3341215.3356980) (Yang and Druga, 2019) | Is a Unique construction toy with inflatable pieces. The set includes silicone pieces with two LEGO pieces on two sides and an air pump to inflate the inflatable silicone bricks. Different silicone pieces make different shapes when inflated such as I’d, S’s, and C’s. Essentially the same as a LEGO-type brick toy, same size pieces, with the addition of inflatable bricks. |
| DIY Model [Doll House](https://www.hepsiburada.com/pershang-ahsap-esyali-villa-ev-3-boyutlu-yapboz-p-HBV00000VMNGH) | A more literal construction set compared to block-type toys. A set of thin wooden pieces, strict instructions to construct doll houses, etc. Repetitive play experience that doesn’t allow creative input. |
| [Marble Maze](https://www.amazon.com/marble-maze/s?k=marble+maze) | This construction toy comprises various translucent pieces designed to create a maze. Some pieces are tubes stacked on top of each other; the rests are slides and stairs that can be attached to the tube pieces so that marble can run through. The marble is placed on the top of the building, makes its way down through the tubes, and slides all over the bottom. Beneficial for learning about movement and gravity. It can be adapted to large scale as well. |
| [Rigamajig](https://casholman.com/rigamajig/) | A large-scale construction set made of modules, some circular, some cubic, etc., modeled after archetypal playground elements, and children are encouraged to build their playground. The modules can be stacked on top of each other or attached using connectors that fit into the holes in the building blocks. Since the sizes of the blocks are large, children can walk through their constructions and experience their build. |
| [Imagination Playground](https://casholman.com/imagination/) | A large-scale construction set made of foam blocks, some circular, some cubic, etc., modeled after archetypal playground elements, and children are encouraged to build their playground. The modules can be stacked on top of each other or attached using connectors that fit into the holes in the building blocks. Since the sizes of the blocks are large, children can walk through their constructions and experience their build. |
| Gigi [Blocks](https://gigibloks.com/pages/gallery) | Large cardboard blocks with tabs on the top and gaps underneath, like LEGO, aside from the size. These cardboard bricks can be stacked on top of each other to build house-like structures. |
| [Habitadule](https://www.youtube.com/watch?v=swSaCj53UeY) | Large scale construction toy consisting of large square cardboard and smaller circles and semicircles. The circular pieces have slits at different angles that fit the slits in the square units and act as connectors. They allow the squares to be attached at different angles, allowing children to build playgrounds or house-like structures they can walk through. |
| [The Toy](https://www.google.com/search?q=Ray+Eames+The+Toy&rlz=1C1GCEU_trTR872TR872&sxsrf=ALiCzsaO6iVzJYUu5V7LBkTj8XeLbslMQw:1665569337800&source=lnms&tbm=isch&sa=X&ved=2ahUKEwiVoumludr6AhUmQPEDHQFFD2gQ_AUoAXoECAEQAw&biw=1536&bih=754&dpr=1.25) (Ginoulhiac, 2013) | Is a Large-scale construction set made of fiberglass sticks and 30-inch triangle and square panels made of vinyl. It can be used for constructing anything from tents to houses to tunnels. |
| [Sifteo](https://en.wikipedia.org/wiki/Sifteo_Cubes) (Geurts et al., 2014) | A sifteo cube is a square device with a 1.5-inch touch screen. Multiple cubes are synced for a unique video game experience. The cubes become a part of a single game. They are moved, tilted, and rearranged to solve puzzles and play games. The play experience is balanced between tangible user interfaces and typical block play. |
| [Co-gnito](https://doi.org/10.1145/3527927.3532803) (Panagiotidou et al., 2022) | Is An advanced urban mental mapping game played on a board made of square tiles. The tiles can be added, removed, or replaced depending on the story being told by the participant. The players create a map of a prior spatial experience on the board using the “path”, “node”, “edge”, “landmark,” and “district” pieces that they place on the board. |
